# Improving Natural Enemy Selection in Biological Control through Greater Attention to Chemical Ecology and Host-Associated Differentiation of Target Arthropod Pests

**DOI:** 10.3390/insects13020160

**Published:** 2022-02-02

**Authors:** Morgan N. Thompson, Raul F. Medina, Anjel M. Helms, Julio S. Bernal

**Affiliations:** 1 Department of Entomology, Texas A&M University, College Station, TX 77840, USA; mthompson@tamu.edu (M.N.T.); rfmedina@tamu.edu (R.F.M.); amhelms@tamu.edu (A.M.H.); 2 Ecology and Evolutionary Biology Program, Texas A&M University, College Station, TX 77840, USA

**Keywords:** biological control, host-associated differentiation, chemical ecology, insect pest management

## Abstract

**Simple Summary:**

Some species of insect herbivores can feed on a wide variety of plant species. Over evolutionary time, insect herbivores can associate preferentially with different host-plant species, which frequently leads to genetic divergence between populations of an herbivore species. This phenomenon, referred to as host-associated differentiation (HAD), not only affects insect herbivores, but can also impact their associated natural enemies, particularly predatory or parasitic insects, which are responsible for biological control in agroecosystems. Although the mechanisms underlying HAD in populations of herbivores and associated natural enemies remain underexplored, we argue that the chemical communication between plants, herbivores, and natural enemies likely plays a major role. Chemical cues emitted by plants and insect herbivores influence natural enemy foraging behavior, and divergent chemical cues can lead to natural enemy HAD, ultimately shaping biological control. In this synthesis paper, we explore how the chemical ecology of HAD could influence classical, conservation, and augmentative biological control, and propose research agendas to further biological control efficacy through natural enemy selection.

**Abstract:**

Host-associated differentiation (HAD) refers to cases in which genetically distinct populations of a species (e.g., herbivores or natural enemies) preferentially reproduce or feed on different host species. In agroecosystems, HAD often results in unique strains or biotypes of pest species, each attacking different species of crops. However, HAD is not restricted to pest populations, and may cascade to the third trophic level, affecting host selection by natural enemies, and ultimately leading to HAD within natural enemy species. Natural enemy HAD may affect the outcomes of biological control efforts, whether classical, conservation, or augmentative. Here, we explore the potential effects of pest and natural enemy HAD on biological control in agroecosystems, with emphases on current knowledge gaps and implications of HAD for selection of biological control agents. Additionally, given the importance of semiochemicals in mediating interactions between trophic levels, we emphasize the role of chemical ecology in interactions between pests and natural enemies, and suggest areas of consideration for biological control. Overall, we aim to jump-start a conversation concerning the relevance of HAD in biological control by reviewing currently available information on natural enemy HAD, identifying challenges to incorporating HAD considerations into biological control efforts, and proposing future research directions on natural enemy selection and HAD.

## 1. Introduction

Symbiotic associations can influence speciation and generate biodiversity [1,2,3]. These associations encompass a wide range of interspecies interactions, from mutualism to commensalism to parasitism. For parasitism, coevolution between host and parasite results in highly specific interactions that promote parasite access to host resources [4]. Parasites adapt to evade or manipulate the immune system of specific host species for their own benefit [5]. Host specialization is common for parasitic insects, including natural enemies of herbivorous insects, such as parasitoids, which typically evolve to evade immune responses of specific insect hosts [6,7,8]. Functionally, insect herbivores are parasites of the plants they feed upon. Most herbivores specialize on a narrow range of host plants [9,10,11,12], evolving mechanisms to detoxify, suppress, or avoid host-plant defenses [13]. For both herbivores and their natural enemies, host detection and acceptance during foraging are critical processes for the initiation of parasitism, and both processes are mediated by chemical cues [14]. Parasites develop heightened capacities over evolutionary time to recognize highly specific, host-associated chemical cues, allowing them to distinguish host from non-host, which drives the evolution of host specialization.

Despite the prevalence of host specialization in parasitic insects [8,9,15], host shifts often occur and lead to the exploitation of new host species [16]. Host shifts can provide enemy-free space [17], reduce intra- and interspecific competition [18], and even increase access to nutrients [19]. In the most extreme cases, genetically divergent populations within a species can evolve to preferentially occur on different host species, and this could represent an initial step towards speciation. This phenomenon is referred to as host-associated differentiation (HAD) [20,21,22]. HAD is the product of reproductive isolation occurring over evolutionary time as individuals mate nearly exclusively with conspecifics associating with the same host. Host-associated mating exacerbates genetic differences between populations due to selection, genetic drift, and mutation. Due to their relatively short generation times, some insects may rapidly develop HAD [23], with evidence suggesting that insects can evolve to exploit novel hosts within as little as 20 years (equivalent to 40 generations) [24]. Diverging host use across populations requires the evolution of differences in multiple traits related to detecting and responding to novel host-associated chemical cues [25]. In agroecosystems, HAD can influence populations of herbivorous insect pests and their associated natural enemies [23]. Understanding the ecological factors underlying HAD in agroecosystems could inform biological control implementation. Here, we review how chemical ecology can help to delineate the mechanisms underlying natural enemy HAD, and discuss implications for biological control.

Although geographic isolation of populations associated with different hosts can contribute to evolution of HAD, most of the available evidence points to the occurrence of HAD among populations living in sympatry [26]. Sympatry indicates that gene flow can occur between divergent populations but HAD reduces gene flow due to preferential mating between individuals associating with the same host. For instance, sympatric pea aphid (*Acyrthosiphon pisum*) populations exhibit HAD across host plants that frequently co-occur in nature [27], and since pea aphids exclusively mate on host plants, host choice correlates with assortative mating between host-associated individuals and structures genetic divergence among pea aphid populations [28,29,30]. A number of factors may contribute to HAD evolution and maintenance in populations living in sympatry. First, host-associated populations are linked to host phenology and if phenology varies across host species, this can result in resource acquisition at different times and thereby restrict mating to the same host-associated individuals. For example, two sister species of live oak (*Quercus virginiana* and *Q. geminata*) exhibit distinct budbreak phenology, which drives HAD in a gall-forming wasp species despite sympatry in the corresponding wasp populations [31]. Second, feeding mode may drive HAD evolution, and endophagous feeding, such as by gall formers and leaf miners, appears to more frequently lead to HAD compared to other feeding modes [23]. Third, recent colonization of novel host species can promote HAD, particularly if novel hosts are biologically similar to hosts already exploited. For example, potato (*Solanum tuberosum*) was domesticated in South America, and following the introduction of potato to North America, Colorado potato beetle (*Leptinotarsa decemlineata*) expanded its host range to feed on potato. Prior to the introduction of potato, Colorado potato beetle was already pre-adapted to exploit solanaceous plants, illustrating how pre-adaptation can contribute to HAD establishment [32]. Fourth, host fidelity, or the tendency of adults to return to the same host species used during early development, can also contribute to HAD [33,34]. For insects, host fidelity generally equates to females ovipositing on the same host species they consumed as immatures. Although the factors described here are not an exhaustive list of all possible scenarios leading to HAD in sympatry, each highlighted factor requires differentiated herbivore populations to evolve unique host location and acceptance traits, and these traits correspond predominantly to detection of divergent chemical cues.

For the HAD of insect herbivores, associating with different host-plant species requires evolving the capacity to discriminate between semiochemicals produced by divergent host plants or herbivore populations. Semiochemicals are broadly classified as chemical signals or cues emitted by organisms that elicit responses in receiving organisms: when receivers and emitters are within the same species, the semiochemical is a pheromone, whereas if they are of different species, the semiochemical is an allelochemical (e.g., kairomone). Thus, pheromones communicate messages to members of a same species, and for insect herbivores, aggregation pheromones indicate the location of a suitable host plant to conspecifics. Although pheromone emissions are typically thought to be under strong selective pressure, there are instances in which pheromone emissions diverge within a species, such as aggregation pheromones across two distinct populations of western pine beetles [35]. Divergence in pheromone emissions within an herbivore species may correspond to populations associating with different host plants. In such cases, conspecifics responding to pheromone emissions of a host-associated population drive assortative mating and further differentiate populations [36].

Similar to pheromones, kairomones can reveal the presence of a suitable host plant to herbivores, thus modifying herbivore behavior, and likely promoting herbivore HAD. Plant volatiles or other secondary metabolites convey the identity and suitability of a host plant to foraging herbivores. After locating a suitable host, herbivore feeding triggers the production of herbivore-induced plant volatiles (HIPVs), which are specific to the identity of the attacking herbivore [37]. Foraging herbivores can detect HIPVs and use these cues to assess the acceptability of a host plant [38,39,40]. During the early evolution of HAD, divergent populations of herbivores presumably adapt to recognize and respond to different host-plant chemical cues. For instance, host-associated pea aphids are stimulated by chemical cues from their respective host plants, indicating that host-plant semiochemicals promote herbivore HAD through attraction towards hosts rather than repellence from non-hosts [41]. Similarly, host-plant primary and secondary metabolites strongly differ across plant species attacked by South American fruit flies (*Anastrepha fraterculus*) that exhibit HAD [42]. While this points toward host-plant cues as drivers of HAD, male pheromone emissions are also divergent and contribute to fruit fly HAD [42], highlighting the complex reality that host- and conspecific chemical cues may simultaneously underpin HAD.

Natural enemy populations may also be subject to the evolution of HAD because they typically live in close association with their herbivorous hosts. For example, the evolution of HAD in herbivore host species may drive co-diversification of associated natural enemies, which is termed “sequential radiation” [43]. Apple maggot (*Rhagoletis pomonella*) and its associated parasitoids is the best studied example of natural enemy HAD in agricultural systems [44,45,46]. Herbivorous apple maggots use fruits for mating and oviposition, and populations show HAD between apple (*Malus domestica*) and hawthorn (*Crataegus* spp.) hosts. Apple maggot HAD is maintained in part by host fidelity through the attraction of apple maggots to natal fruit volatiles over non-natal fruit volatiles. Apple maggot HAD dictated the co-diversification, and the resulting natural enemy HAD of three species of apple maggot-specific parasitoids [47]. Interestingly, those natural enemies also exhibit strong preferences for fruit volatiles from their natal hosts, and avoid non-natal host volatiles [47]. Reciprocal rearing experiments for one parasitoid wasp species, *Diachasma alloeum*, further demonstrated a genetic basis for volatile preference, indicating that attraction to these cues is a trait under divergent selection across *D. alloeum* populations [47]. Relatedly, because HIPVs are released exclusively upon herbivory and attract natural enemies to host plants to eliminate herbivores, they serve as consistent and reliable foraging cues, and likely underline many instances of natural enemy HAD. For example, a predator of the gall-forming fly *Eurosta solidaginis* shows preferences for natal HIPVs relative to non-natal HIPVs [48], which given that *E. solidaginis* exhibits HAD across two *Solidago* plant species, indicates that divergent HIPVs can underlie sequential radiation of natural enemies.

In addition to plant volatiles, natural enemies can locate prey by eavesdropping on semiochemicals emitted directly from prey, such as pheromones or kairomones (i.e., metabolic byproducts). For example, upon perceiving predation risk, many aphid species emit (*E*)-β-farnesene as an alarm pheromone to warn conspecifics, but (*E*)-β-farnesene also attracts many species of natural enemies [49]. Herbivore divergence in pheromone emissions following HAD could therefore impact natural enemy foraging, and over time drive sequential radiation of associated natural enemy populations. Upon locating prey through semiochemical detection, prey selection by natural enemies is often driven by prey toxicity [50]. Some herbivores sequester secondary metabolites from host plants for defense against natural enemies, such as Western corn rootworm (*Diabrotica virgifera virgifera*) larvae sequestering benzoxazinoids for protection from belowground natural enemies [51]. As an herbivore evolves HAD upon colonizing a novel host plant it may sequester novel secondary metabolites that could impact its natural enemies, and lead to sequential radiation.

**Figure 1:**
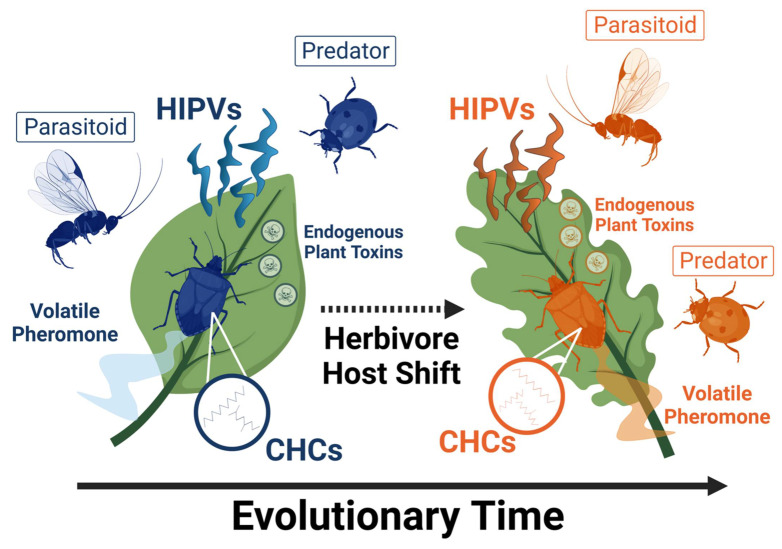
Conceptual model showing one possible evolutionary route to sequential radiation or natural enemy host-associated differentiation (HAD) following herbivore HAD. Populations of herbivores and natural enemies associating with different host-plant species represent genetically divergent populations within the same species of herbivore and natural enemy. HIPVs = herbivore-induced plant volatiles; CHCs = cuticular hydrocarbons. Figure generated using BioRender.

Importantly, sequential radiation is not always evident, and there are several examples of insect pest HAD that seemingly do not extend to associated parasitoids (Table 1). Currently, all examples of natural enemy HAD follow demonstration of host HAD, but it is important to note that natural enemy HAD may occur in certain systems independently of host HAD [52]. Strong intraspecific competition, for instance, may drive divergence in host use in a given natural enemy species, regardless of HAD in its herbivore host [43]. In such cases, herbivorous pests may benefit from enemy-free space on certain host plant species relative to others, and variation in HIPVs would likely underlie natural enemy divergence in host use. Nevertheless, we focus our review primarily on sequential radiation, or the co-diversification of associated natural enemies as herbivore hosts evolve HAD (Figure 1), as we predict it is likely that natural enemy HAD strongly influences biological control in agroecosystems.

Optimal selection of natural enemies is fundamental for effective biological control in agroecosystems. For example, invasive pest species are often combated through classical biological control, which typically relies on discriminating among a suite of natural enemies originating in the invasive pest’s native range, and selectively releasing the most promising natural enemy species in the invaded range. Similarly, conservation and augmentative biological control efforts are enhanced by deliberate selection of natural enemies to conserve or release in agroecosystems. Across all biological control strategies, natural enemy selection often occurs at the species level, and efforts to determine any non-target effects usually focus on interspecies interactions, such as predation or parasitism of non-pest herbivores or predation of other natural enemies, i.e., intraguild predation [53,54]. Within-species variation, in contrast, is often neglected in natural enemy selection, or historically has been restricted to broad-scale geographic or climate-driven variation (e.g., [55]). Although not sequential radiation, an early example illustrates how within-species variation can affect natural enemy foraging preference and biological control outcomes. In the 1930s, a “Japanese race” of the parasitoid *Comperiella bifasciata* was imported to California for biological control of invasive Yellow scale (*Aonidiella citrina*) on citrus, and rapidly provided effective control of the scale in southern California [56]. Subsequently, in the 1940s, a “Chinese race” of the parasitoid was introduced to control Yellow scale in the San Joaquin Valley. Soon after, biological control of Yellow scale was achieved, though later it was found that control was due to the “Japanese race,” which had been colonized earlier, though reportedly without success. Subsequent field and laboratory studies demonstrated that the “Japanese race” was exclusively parasitic on Yellow scale, while the “Chinese race” was parasitic on California red scale (*Aonidiella aurantii*) and Yellow scale, but performed better and preferred the former host, and became an important component of its biological control in California [56]. Clearly, a better understanding –at the time– of how parasitoid preferences for different host species may vary among distinct parasitoid populations would have ultimately precluded the colonization of the “Chinese” race in the San Joaquin Valley. Thus, the presence of genetically distinct host-associated parasitoid populations may play a key and traditionally overlooked role in biological control, whether classical, conservation, or augmentative strategies are pursued. Here, our main objective is to identify ways in which consideration of HAD may improve natural enemy selection—an enduring, critical issue in biological control, propose broad research directions to further our understanding of chemical cues relevant to HAD and natural enemy selection, and stimulate a discussion concerning the relevance of HAD in biological control.

**Table 1:** Studies testing for sequential radiation in natural enemies, or natural enemy HAD in a single herbivore species which differentially associates with two host-plant species. To be included in this table, quantification of genetic difference between natural enemy populations was required. The genetic signature of HAD typically corresponds to F_ST_ ≥ 0.15 between sympatric, host-associated populations.

Plant Species	Herbivore Species	Natural Enemy Species	Natural Enemy HAD Detected?	Source
Alfalfa (*Medicago sativa*) Red Clover (*Trifolium pratense*)	Pea Aphid (*Acyrthosiphon pisum*)	Parasitoid (*Aphidius ervi*)	No	[57]
Pecan (*Carya illinoinensis*) Water Hickory (*Carya aquatica*)	Yellow Pecan Aphid (*Monelliopsis pecanis*)	Parasitoid (*Aphelinus perpallidus*)	No	[58]
Almond (*Prunus dulcis*) Apricot (*Prunus armeniaca*) Peach (*Prunus persica*) Plum (*Prunus domestica*)	Aphids (*Hyalopterus* spp.)	Parasitoid (*Aphidius transcaspicus*)	No	[59]
*Pinus nigra* *Pinus sylvestris*	Pine Processionary Moth (*Thaumetopoea pityocampa*)	Specialist Parasitoid (*Baryscapus servadeii*) Generalist Parasitoid (*Ooencyrtus pityocampae*)	No	[60]
Tall Goldenrod (*Solidago altissima*) Giant Goldenrod (*Solidago gigantea*)	Gallmakers (*Rhopalomyia solidaginis*, *Gnorimoschema gallaesolidaginis*)	Parasitoids (*Platygaster variabilis*, *Copidosoma gelechiae*)	Yes	[61]
Apples (*Malus domestica*) Hawthorns (*Crataegus* spp.)	Apple Maggot (*Rhagoletis pomonella*)	Parasitoids (*Diachasma alloeum*, *Utetes canaliculatus, Diachasmimorpha mellea*)	Yes	[47]
Yuccas (*Yucca* spp.)	Bogus Yucca Moths (*Prodoxus* spp.)	Parasitoids (*Eusandalum* spp.)	No	[62]
*Acacia* spp.	Thrips (*Kladothrips* spp.)	Kleptoparasitic Thrips (*Koptothrips dyskritus, Koptothrips flavicornis*)	Yes	[43,63]

## 2. Host-Associated Differentiation: Challenges for Biological Control Programs

### 2.1. Classical Biological Control

Natural enemy selection remains among the most critical issues to resolve for classical biological control. This biocontrol strategy targets invasive pests, and relies on foreign exploration for natural enemies in an invasive pest’s native range. These natural enemies are then released into the invaded range, where they become established, and may provide long-term suppression of invasive pest populations. In recent decades, increasing concern for unanticipated ecological impacts of natural enemies released for classical biological control has led to greater scrutiny and deliberate selection of natural enemies for release that pose the least ecological risk, while showing the greatest promise for effective pest suppression [64,65,66]. While there are numerous examples of classical biological control successes, such as control of cottony cushion scale (*Icerya purchasi*) with the Vedalia beetle (*Novius cardinalis*) in California citrus orchards, there are many failed efforts, including a few cases in which non-target effects or ecological impacts have been documented [67,68,69]. To best safeguard against unwanted effects from natural enemy introduction, a detailed understanding of natural enemy biology and interactions with novel ecological communities is required. For example, introduced natural enemy species may expand their host range upon release in a novel environment, and although this is a problem regardless of natural enemy HAD, the attack of non-target species could lead to the evolution of HAD on newly adopted host species. Such host range expansions, especially if they lead to the evolution of HAD on novel hosts, could harm classical biological control efforts if the populations of novel hosts are measurably suppressed or if those species are protected or prized.

When searching for natural enemies in native ranges, incorporating natural enemy HAD into evaluations of candidate species would enhance classical biological control efforts. Traditionally, host species specificity, as well as matching climates between introduced and native ranges, were some of the major criteria used in natural enemy selection [55,70]. While these factors remain important considerations, affirmation that pest species exhibit HAD on different plant species in the native range would necessitate considering whether natural enemies display HAD, i.e., sequential radiation. Such sequential radiation may lead to differential natural enemy preference for HIPVs from different host plants or other host-associated chemical cues. For instance, in the Americas, the generalist pest species, fall armyworm (*Spodoptera frugiperda*) comprises at least two sympatric and morphologically identical, yet genetically distinct strains, which associate preferentially with different species of host plants [71]. Previous research on a larval parasitoid of fall armyworm detected different host preferences for the two known fall armyworm strains, the *Rice* and *Corn* strains [72]. To control fall armyworm populations in specific crops in recently invaded regions, selection of non-corresponding host-associated parasitoids would likely yield diminished classical biological control, although it is important to note that other control methods used in conjunction with biological control would likely improve the management of fall armyworm. Classical biological control of fall armyworm strains highlight how the consideration of herbivore HAD and natural enemy sequential radiation can improve natural enemy selection processes.

Deliberate or unwitting consideration of HAD in invasive pest species, even if HAD is undocumented or unsuspected, may also reduce the frequency of biological control failures. For instance, the “Chinese race” of *C. bifasciata* plausibly would not have been considered for release against Yellow scale if its preference for- and enhanced performance on California red scale were known in advance (see above). Recently, prior to searching for parasitoids for classical biological control of the mealybug *Delottococcus aberiae*, an invasive pest of citrus, Beltrà et al. (2015) studied the pest’s host affinities [73]. Through integrative taxonomy (i.e., combined morphological and molecular characterization) they uncovered divergent populations of *D. aberiae* on citrus, wild olive, and *Chrysanthemoides monilifera* from several South African provinces. They found that the invasive *D. aberiae* populations from Spanish citrus orchards clustered together with populations on citrus from a single South African province, with which they shared COI haplotypes. Based on this result, Beltrà et al. recommended that *D. aberiae* parasitoids be sought specifically on citrus and exclusively in the South African province they identified in their study [73]. Such parasitoids likely respond to citrus HIPVs or other semiochemicals produced by the citrus-associated *D. aberiae* population. Obtaining parasitoids from other *D. aberiae* populations, such as those associated with wild olive or *Chrysanthemoides*, would likely result in poorer biological control as these parasitoids would not be adapted to respond to citrus-associated semiochemicals from plants or insect hosts.

Even when considering HAD in herbivore or natural enemy populations, challenges remain when invasive herbivores expand their diet breadth in introduced ranges. Upon introduction into new geographic locations, invasive herbivores often colonize novel host plants [74,75], as seen in the northeastern United States with the brown marmorated stink bug (*Halyomorpha halys*), and more recently with the spotted lanternfly (*Lycorma delicatula*). Both species display impressive capacities to feed on wide varieties of host plants, ranging from fruit and vegetable crops in diverse plant families to woody gymnosperms [76,77]. As these invasive herbivores expand their diets, natural enemies introduced from their native ranges may struggle with locating hosts on novel plants [78], reducing biological control effectiveness. One possible explanation is that novel host plants may not perceive invasive insect herbivory, instead responding only to mechanical wounding and not emitting the particular HIPVs essential for effective natural enemy foraging [78]. Disruption of host-associated chemical cues could reduce classical biological control efforts. Rapid expansion in diet breadth indicates that invasive herbivores may outpace natural enemies in HAD as they move to novel plants and evade natural enemy attack. Fall armyworm, which has numerous hosts across many plant families [79], may illustrate this case as it continues to spread across continents and encounters novel hosts. Overall, however, very few empirical studies to date have characterized HIPVs following invasive insect herbivory, which is a critical gap in our knowledge and could contribute to our understanding of natural enemy sequential radiation.

Evolutionary or breeding history of novel crop hosts is also important to consider when invasive herbivores expand their diet breadth. In the case of fall armyworm, maize—the most affected crop in invaded regions of Africa and Asia—had not been exposed to fall armyworm for hundreds of crop and insect generations, so its HIPV emissions, as well as other defensive responses, may have been lost or weakened. In this way, fall armyworm associations with maize in Africa and Asia could be considered ‘novel.’ Recently, one study showed that North American maize breeding lines did not emit the HIPV (*E*)-β-caryophyllene, known to be emitted in response to leaf-feeding by *Spodoptera littoralis*, and root-feeding by Western corn rootworm [80]. The same study showed that the gene responsible for the HIPV’s emission, *TPS23*, is maintained by positive selection in European maize and its wild ancestor, but is largely inactive in most North American lines, which do not emit the HIPV. The combined results of that study and a recent study [Bernal et al. unpublished] suggest that (*E*)-β-caryophyllene signaling was lost during the breeding of North American maize lines in the last ~100 years. The loss of (*E*)-β-caryophyllene has been shown to disrupt attraction of insect-killing nematodes to Western corn rootworm-infested roots, and likely also disrupts natural enemy attraction to fall armyworm feeding on such lines. Variation in HIPV emissions within plant species highlights another important source of variation that can influence natural enemy attraction to host plants and ultimately shape natural enemy HAD.

In addition to HIPVs, some species of natural enemies can detect prey pheromones and use pheromone emissions to locate prey when foraging. Natural enemies can respond to prey sex pheromones, as is the case for *Elater ferrugineus*, the predator of the scarab beetle *Osmoderma eremita* [81]. Prey aggregation pheromones also attract natural enemies of thrips (*Frankliniella occidentalis*) [82], stink bugs (*Thyanta pallidovirens*) [83], and bark beetles (*Ips* spp. and *Dryophthorus americanus*) [84]. In some instances, the chemical blend of prey pheromone emissions can diverge within a species and produce unique ‘pheromone strains’ of pestiferous herbivores [85]. For example, the European corn borer (*Ostrinia nubilalis*), a major pest of maize worldwide, consists of two distinct strains in which females produce different sex pheromones, driving assortative mating within each population [86]. Studies of European corn borer host-plant associations in France revealed that the different strains preferentially colonized different host plants, suggesting that HAD could play a role in maintaining the distinct strains [86]. The divergent pheromone blends or host-plant associations may drive natural enemy HAD in the European corn borer system, but this remains unknown. Intriguingly, a tachinid parasitoid (*Trichopoda pennipes*) is attracted to green stink bug (*Nezara viridula*) aggregation pheromone, and pheromone strains of green stink bug were identified from a failed biological control effort with this natural enemy [87]. An introduction of this parasitoid species from the southern United States to Hawaii resulted in no parasitism of green stink bugs [88], and further investigations revealed divergent pheromone blends between the two stink bug populations [89]. Although these differences were likely driven by geographic isolation rather than green stink bug HAD, the impacts on natural enemy foraging efficiency highlight how changes in chemical cues can disrupt biological control.

Contact pheromones, or cuticular hydrocarbons (CHCs), also mediate within-species recognition among insects. CHCs are primarily made up of various long-chain waxes, such as alkanes and alkenes, and are perceived by physical contact between individual insects and provide relevant information about the identity of a species [90]. The composition of CHCs for herbivores is dictated in large part by diet, as ingested host-plant lipids, proteins, and carbohydrates provide the basis for biosynthesis of CHCs [91]. Intraspecific variation in CHC profiles can occur when individuals from a given herbivore species feed on different host-plant species [92,93]. Host-plant-induced variations in CHCs can drive mating preferences for individuals sharing the same host plant and ultimately result in assortative mating within populations [94], suggesting CHCs play an important mechanistic role in the beginning stages of HAD. For example, CHC profiles of mustard leaf beetles (*Phaedon cochleariae*) vary depending on host-plant species use, and these changes in CHCs determine mating preferences, as individuals prefer to mate with other individuals from the same rather than different host plants [95,96,97]. Some natural enemy species, such as parasitoids, also rely on CHCs to identify their host species for oviposition [98,99]. Although only one study to date has connected prey HAD to changes in CHCs [100], we postulate that this is likely a common scenario, and the extent to which CHCs diverge within prey populations could determine whether natural enemy HAD will also occur. Divergent pheromone emissions could play roles in natural enemy HAD and efficacy, which should be considered in efforts for natural enemy encouragement in classical biological control.

### 2.2. Conservation Biological Control

Conservation biological control utilizes natural enemy populations already present in agroecological landscapes to suppress pests on crops, increasing the abundance of natural enemies and enhancing the impact of biological control on pest populations [101]. Accomplishing pest suppression using this strategy typically involves habitat manipulation within agricultural fields or surrounding areas. For instance, cover crops, intercropping, and planting floral borders all enhance structural complexity and increase levels of nutritional resources necessary for natural enemy foraging and survival [102]. In addition to habitat manipulation, manipulation of chemical cues to enhance natural enemy attraction to agricultural fields is another conservation biological control tactic that shows promise [103,104]. Many natural enemies show strong attraction to the same HIPVs, such as methyl salicylate, and efforts to deploy synthetic methyl salicylate lures have yielded generally positive results in agricultural fields by enhancing natural enemy recruitment and arrest [105]. Despite advances in habitat and chemical manipulation to enhance conservation biological control, much work remains in furthering the use and efficacy of conservation biological control. The chemical ecology of natural enemy HAD may play an overlooked role in advancing conservation biological control practices.

Although conservation biological control can contribute to suppression of invasive pests, this biocontrol strategy is most important for native pests as natural enemies are presumed to be adapted to pests with which they have been long associated over novel hosts or prey, such as invasive pests. Extensive co-evolutionary relationships in native ranges between natural enemies and prey results in closely adapted natural enemy communities [106], which readily detect and respond to chemical cues from herbivores and their infested plants. Non-native host plants, such as introduced crops, however, could impact conservation biological control if native pests expand their diet breadth to introduced hosts. Agricultural crops are a recurring type of non-native plant, as crops are often grown throughout the world in areas far from their regions of origin [107]. As such, any colonization event by an herbivore in its native range to an introduced crop represents a novel, non-co-evolved interaction. For example, two species of pestiferous, stem boring moths (*Diatraea* spp.) are co-evolved with maize but host-shifted to introduced agricultural crops of the maize (Poaceae) family—sugarcane, sorghum, and rice—, and genetically distinct populations of the moths were shown to attack each crop species [108]. Further, investigations into associated parasitoids revealed low levels of parasitism in the introduced crops relative to maize, particularly for one of the moth species [108]. A plausible explanation was that introduced crops did not produce attractive HIPVs for native natural enemies, creating “enemy-free space” for the stemborers, which allowed them to effectively hide from their associated predators or parasitoids. Pest HAD with novel crops could serve as a mechanism for pest emergence, and stymie conservation biological control efforts at their outset.

Beyond introduced crops, native pests can also colonize introduced or invasive non-crop species with the potential for HAD to develop over time and alter conservation biological control [109]. Host range expansions to novel plants can provide “enemy-free space” for herbivores and thereby increase their survival and population sizes. For instance, in the presence of predators, the Alaskan swallowtail butterfly (*Papilio machaon aliaska*) adopted novel hosts on which larval survival increased relative to ancestral hosts [110]. However, when predators were removed from the system, this effect was reversed and larval survival was lower on novel hosts than ancestral hosts, suggesting novel hosts are lower quality but offer greater protection from natural enemies than ancestral hosts [110]. These findings underscore how the incorporation of a novel host into the diet breadth of a native herbivore may disrupt conservation biological control. Unfamiliar host-associated chemical cues likely underlie natural enemy avoidance or the non-detection of native prey on novel hosts. For example, the non-detection of native prey on novel hosts may occur if novel host plants do not emit HIPVs that are attractive to native natural enemies. Recent evidence from ragwort (*Jacobaea vulgaris*) plants found that native populations emitted high levels of HIPVs and readily attracted native natural enemies, whereas invasive ragwort populations produced lower levels of HIPVs [111]. The same species of natural enemies are not present in the invasive range of ragwort, leading invasive ragwort populations to reduce investment in HIPV emissions [111]. In the context of the invaded ecological community, invasive plants offer native herbivores the opportunity to avoid detection by their co-evolved natural enemies through reduced HIPV emissions. Avoidance of natural enemies may promote native herbivore host shifts to novel host plants [112]. Relatedly, native herbivores may sequester secondary metabolites from novel plants and increase or decrease their toxicity to native natural enemies [113,114]. Such changes in prey toxicity can cascade to help or hinder natural enemy attack or development within hosts [115,116]. For instance, the performance of a native parasitoid species (*Cotesia glomerata*) was reduced when completing development in herbivores feeding on a novel crucifer species relative to native *Brassica nigra* [117], and it is possible that differences in secondary metabolite sequestration by native herbivores underlie differences in natural enemy performance [109]. If herbivore toxicity to natural enemies increases when herbivores feed on novel hosts, this could favor herbivore HAD, if sufficient evolutionary time elapsed and survival increased on novel host plants in the presence of natural enemies. Regardless of the mechanism, native herbivore HAD on novel, introduced or invasive host plants can hamper conservation biological control.

Invasive insect herbivores are generally not the targets of conservation biological control. However, how invasive herbivores interact with native ecological communities can determine the outcomes of chemically mediated predator-prey interactions and conservation biological control. For instance, cabbage white (*Pieris brassicae*) herbivory on its native host plant (*Brassica rapa*) induces HIPVs that recruit the braconid wasp parasitoid *C. glomerata*, but this recruitment stops when plants are dually attacked by both cabbage whites and an exotic herbivore, *Spodoptera littoralis* [118]. Similarly, when an invasive brown marmorated stink bug concurrently attacks plants with native herbivores, the natural enemy attraction to native HIPVs is attenuated [119]. Thus, invasive herbivores can have far-reaching effects in ecological communities, ultimately interfering with conservation biological control through disrupted HIPV recruitment of natural enemies [120]. To date, most research on HAD and sequential radiation has focused on pairwise interactions between herbivores and their associated natural enemies, neglecting the role of other members of the ecological community in disrupting chemical cues used for host location. Expanding our view of HAD to entire ecological communities will likely shape our understanding of the factors driving the development of sequential radiation [121]. Further explorations into how invasive species mediate interactions between natural enemies and their herbivore hosts, particularly in the context of sequential radiation, will enhance conservation biological control.

### 2.3. Augmentative Biological Control

In the case of augmentative biological control, which utilizes natural enemies reproduced in laboratories or industrial facilities for release against pests, HAD may be most important in rearing facilities, in reference to both natural enemy and host or prey species. Rearing environments are divergent from natural and agricultural habitats, particularly because they typically are designed to enhance production (quantity or volume) over quality [122]. In artificial rearing environments, competition is managed and limited to intraspecific competition, hosts or prey for natural enemies and food for hosts or prey are readily available, and all other environmental variables are optimized for natural enemy or host survival and reproduction. Thus, the selective pressures in these environments drastically differ from those in natural or agricultural habitats, and select for adaptation to rearing environments; as importantly, genetic drift and inbreeding may also affect mass-reared natural enemy and host population [123,124,125], and contribute to the evolution of HAD. For example, because of selection, drift, or inbreeding, natural enemies may become adapted to laboratory-adapted hosts that differ from field populations of hosts, and such adaptation may lead to natural enemy HAD in the rearing environment, and biological control failures in the field [126]. Similarly, natural enemy HAD may evolve when natural enemies are reared on factitious hosts (i.e., different from the targeted pest) because these can be reared more economically than the targeted pest. Overall, natural enemy HAD on the rearing host can reduce efficacy of biological control in the field, as was demonstrated for the specialist egg parasitoid *Trichogramma galloi* [127]. Rearing this parasitoid for numerous generations on a factitious host resulted in lower parasitoid fitness when reintroduced to the natural, pestiferous host [127], suggesting natural enemy HAD on the factitious host. Similar reductions in natural enemy efficacy following rearing on a factitious host were also observed for natural enemies of boll weevils (*Anthonomus grandis*) [128] and pea aphids [129]. Rearing environments also remove many of the chemical cues used in host location for natural enemies, such as HIPVs, which could result in natural enemy populations that are less responsive to such chemical cues. When rearing natural enemies for augmentative biological control, one possible way to overcome HAD is to frequently introduce natural enemies and hosts from the field to avoid evolution of natural enemy HAD on factitious hosts, and host adaptation to rearing environments [123,125].

## 3. Future Directions

We predict natural enemy and pest HAD influence biological control outcomes under many scenarios. Differential detection of host-associated chemical cues across host populations likely drives natural enemy HAD. Chemical cues derived from plants (e.g., HIPVs) and directly from herbivores (e.g., pheromones) can influence natural enemy foraging and host location, but these chemical cues can change following herbivore HAD. Such changes can cascade to shape herbivore interactions with their associated natural enemies, and possibly even drive sequential radiation. To date, relatively few cases of sequential radiation have been documented, and so far, tests for natural enemy HAD have only been conducted for parasitoids and not for other types of natural enemies, such as predators (Table 1), which could reflect a number of non-mutually exclusive scenarios. First, generalist natural enemies may easily exploit different host species. Relying on a general suite of different hosts rather than one particular host species would likely minimize assortative mating and thereby slow the evolution of natural enemy HAD. Second, during their lifetimes, natural enemies often exhibit phenotypic plasticity in learning. Learning may enhance natural enemy responsiveness to host-associated cues. If natural enemies quickly learn, this could dampen the development of natural enemy HAD, as innate preferences for different hosts would not easily be maintained in such natural enemy populations. Third, it is also possible that natural enemy host-associated populations or host-races are short-lived, evolving relatively quickly into separate species, and reducing the chances of finding true host-races at any given point in time, although this scenario remains difficult to experimentally test. Fourth, natural enemies may not keep up with herbivores as they adopt novel host plants, preventing sequential radiation. To conclusively determine the relevance of any of these scenarios to the evolution of natural enemy HAD, future research should focus on examining natural enemy HAD across more crop-, pest-, and natural enemy associations.

Addressing whether and how frequently natural enemy HAD occurs will improve the predictability of biological control outcomes, whether classical, conservation, or augmentative biological control is pursued. Pest HAD is predicted to occur in endophagous insects with reduced genetic recombination, but there is currently no consensus concerning which natural enemy lifestyles lead more frequently to HAD [23]. Extrapolating from pest HAD predictions, endoparasitoids, particularly those showing haplodiploidy, for example, may be more prone to natural enemy HAD than generalist predators that reproduce sexually, but this has not been explicitly tested. However, some studies show how drift, inbreeding, and selection affect natural enemies in biological control, particularly parasitoids given their haplodiploidy [123,125,130]. Notably, there are examples of HAD that defy theoretical predictions, such as HAD in sexually reproducing species [131], and predators that prefer different host-associated herbivore populations—although the degree of genetic divergence in this natural enemy species has not been elucidated and thereby cannot yet be considered HAD [132]. Without more empirical evidence, it is difficult to make strong predictions, so further investigation into natural enemy HAD across different systems is warranted.

Breeding natural enemies is one way to combat the challenges of HAD in natural enemy selection. Breeding or artificial selection harnesses genetic variation within natural enemy populations to enhance pest suppression and improve biological control [133]. However, identifying natural enemy traits relevant to biological control effectiveness, and with sufficient heritable genetic variation, remains challenging. A number of traits have been proposed, and one of increasing interest is natural enemy learning behavior [133]. Since natural enemies exhibit an impressive capacity to learn and respond to chemical stimuli, natural enemy populations could be artificially selected over many generations for enhanced response to select chemical cues [134]. Such chemical cues would correspond to host-associated herbivores and include plant- or herbivore-derived cues [135]. For instance, the entomopathogenic nematode, *Heterorhabditis bacteriophora*, attacks soil-dwelling insect herbivores, such as the major maize pest, Western corn rootworm. Following belowground rootworm herbivory, maize roots emit (*E*)-β-caryophyllene [136], and this HIPV recruits *H. bacteriophora* to kill rootworms [137]. Previous efforts to employ selective breeding strategies for *H. bacteriophora* increased its responsiveness to (*E*)-β-caryophyllene after only six generations and also enhanced control of rootworms in the field [138]. Employing similar strategies to produce populations of different natural enemies can help to overcome the limitations of biological control for pests exhibiting HAD.

Currently, knowledge of natural enemy HAD extends only to insect predators, parasitoids, or entomopathogenic nematodes. However, microbial entomopathogens are increasingly utilized as biological control agents against insect pests, but it is unknown whether HAD occurs in these entomopathogens. Investigation into a common fungal entomopathogen, *Beauveria bassiana*, revealed no intraspecific genetic variation across 36 different *B. bassiana* strains [139], but it remains unclear if this extends to other species of fungal entomopathogens. Recent research demonstrated sequential radiation between species of tree, beetle, and mutualistic fungal symbiont [140], suggesting a capacity for fungal entomopathogens to also exhibit HAD. Bacterial symbionts or even entire host-associated microbiota can contribute to HAD [141,142,143,144], suggesting a capacity for bacterial entomopathogens to show HAD. The most important bacterial entomopathogen for biological control in agroecosystems is *Bacillus thuringiensis*, or ‘Bt.’ Different subspecies of Bt are utilized to control different types of insects, such as *Bacillus thuringiensis israelensis* for mosquito management or *Bacillus thuringiensis kurstaki* for managing lepidopteran pests, further implying bacterial entomopathogens likely possess the capability to express HAD. Determining the extent of HAD in microbial entomopathogens will improve natural enemy selection.

A final recommendation for future research is to consider pest HAD in the context of pest immune response to biological control agents. Insects deploy immune responses to fend off different types of natural enemies [145]. One common immune strategy against attack from endoparasitoids is encapsulation, often by hemocytes that surround and suffocate parasitoid eggs or young larvae, preventing the development of parasitoids within their host’s body [146]. Encapsulation abilities can vary within species, producing pest strains that are resistant or susceptible to parasitoid attack, as is the case for different strains of *Drosophila melanogaster* attacked by the parasitoid, *Leptopilina boulardi* [147]. Beyond exploiting different host plant species, pests exhibiting HAD may also differ in their immune responses to natural enemies, likely as a result of evolutionary forces (e.g., genetic drift, mutation, selection) acting in unique ways within each host-associated population. In contrast, natural enemies can also show different intraspecific responses to host immune systems. Evidence from recent work with the pestiferous maize stalk borer, *Busseola fusca,* in Africa indicated different subpopulations of a parasitoid species were differentially susceptible to maize stalk borer defenses: one subpopulation evaded host immunity, while another was encapsulated [148]. Natural enemy populations varying in susceptibility to pest immune responses could exacerbate sequential radiation and natural enemy HAD as different natural enemy populations evolve in concert with host-associated prey. Incorporating pest immune responses, particularly in species with already-determined HAD, will likely be important in furthering biological control efforts.

## 4. Conclusions

We conclude that chemical cues underlying HAD are powerful drivers of interactions between host plants, pests, and their associated natural enemies. To improve selection and use of natural enemies in agroecosystems, future work must consider pest and natural enemy HAD, with emphasis on chemical communication among crop plants, pests, and natural enemies. Incorporating investigations into the chemical cues mediating these interactions provides mechanistic links between pest and natural enemy HAD, and offers innovative opportunities to improve natural enemy selection in biological control and manipulate agrochemical ecology to enhance biological control.

## Data Availability

Not applicable.
